# Response of letter to the editor on Procalcitonin: a promising diagnostic marker for sepsis and antibiotic therapy

**DOI:** 10.1186/s40560-017-0260-x

**Published:** 2017-12-06

**Authors:** R. Saikant, Shilpa Ravindran, Ashitha Vijayan, Vani Maya, S. Lakshmi, R. Kartik, Manoj G.

**Affiliations:** Diagnostic Products Division and Cell Culture facility, Corporate R&D Centre, HLL Lifecare Limited, Sreekariyam (P.O), Kerala, Trivandrum, India

## Abstract

In a letter to the editor, Raineri SM et al. have given an insight of another dimension of procalcitonin (PCT) use as a diagnostic tool in invasive candidiasis. But based on our preliminary information, PCT is reported as unconventional modes of diagnosis approach which yet to prove its stand-alone biomarker properties for invasive candidiasis.

## Letter to the editor

In our recent paper in Journal of Intensive Care, we presented a prototypical explanation of procalcitonin (PCT) use as a diagnostic marker in early detection of sepsis and septic shock due to bacterial infections. We concluded that combination of emerging new biomarkers with PCT could be used in terms of good clinical judgement based on which antimicrobial therapy may suggest, thus reducing the prescription and dosage of antibiotic treatment. In addition, we also suggested that PCT alone might not be effective for accurate diagnosis. Our findings in our article are limited to infection-related bacterial sepsis.

In a letter to the editor, SM Raineri and colleagues, suggest that PCT is useful in suspected invasive candidiasis.

As per Raineri SM et al., PCT may also serve as an effective tool for invasive candidiasis [1]. Based on Charles et al. (2006), PCT level is significantly low in patients with candidemia (median 0.65 ng/ml) compared to those with bacteremia (median 9.75 ng/ml). PCT level higher than 5.5 ng/ml demonstrated a 100% negative predictive value (NPV) and 65% positive predictive value (PPV) for sepsis caused by *Candida* spp. [2]. With these findings, we also believe that the level of PCT expression may vary in such conditions. But the limiting factor is that the expression of PCT levels in candidemia is very less in comparison with bacterial infections. Hence, the detection of PCT requires much more sensitive diagnostic methods. A successful biomarker for diagnostic use should be elevated in response to the pathogen at the earliest with detectable threshold quantities. Clearly, more research is needed to realize ‘high certainty of evidence-based recommendations’.

## References

[1] Raineri SM, Cortegiani A, Vitale F, Iozzo P, Giarratano A (2017). Procalcitonin for the diagnosis of invasive candidiasis: what is the evidence?. J Intensive Care.

[2] Charles PE, Dalle F, Aho S, Quenot J-P, Doise J-M, Aube H, Olsson N-O, Blettery B (2006). Serum procalcitonin measurement contribution to the early diagnosis of candidemia in critically ill patients. Intensive Care Med.

